# Protective Effects of Astodrimer Sodium 1% Nasal Spray Formulation against SARS-CoV-2 Nasal Challenge in K18-hACE2 Mice

**DOI:** 10.3390/v13081656

**Published:** 2021-08-20

**Authors:** Jeremy R. A. Paull, Carolyn A. Luscombe, Alex Castellarnau, Graham P. Heery, Michael D. Bobardt, Philippe A. Gallay

**Affiliations:** 1 Starpharma Pty Ltd., Abbotsford, VIC 3067, Australia; carolyn.luscombe@starpharma.com (C.A.L.); alex.castellarnau@starpharma.com (A.C.); graham.heery@starpharma.com (G.P.H.); 2 Department of Immunology and Microbiology, The Scripps Research Institute, La Jolla, CA 92307, USA; mbobardt@scripps.edu (M.D.B.); gallay@scripps.edu (P.A.G.)

**Keywords:** astodrimer, SPL7013, dendrimer, antiviral, SARS-CoV-2, COVID-19, nasal, animal model

## Abstract

Strategies to combat COVID-19 require multiple ways to protect vulnerable people from infection. SARS-CoV-2 is an airborne pathogen and the nasal cavity is a primary target of infection. The K18-hACE2 mouse model was used to investigate the anti-SARS-CoV-2 efficacy of astodrimer sodium formulated in a mucoadhesive nasal spray. Animals received astodrimer sodium 1% nasal spray or PBS intranasally, or intranasally and intratracheally, for 7 days, and they were infected intranasally with SARS-CoV-2 after the first product administration on Day 0. Another group was infected intranasally with SARS-CoV-2 that had been pre-incubated with astodrimer sodium 1% nasal spray or PBS for 60 min before the neutralisation of test product activity. Astodrimer sodium 1% significantly reduced the viral genome copies (>99.9%) and the infectious virus (~95%) in the lung and trachea vs. PBS. The pre-incubation of SARS-CoV-2 with astodrimer sodium 1% resulted in a significant reduction in the viral genome copies (>99.9%) and the infectious virus (>99%) in the lung and trachea, and the infectious virus was not detected in the brain or liver. Astodrimer sodium 1% resulted in a significant reduction of viral genome copies in nasal secretions vs. PBS on Day 7 post-infection. A reduction in the viral shedding from the nasal cavity may result in lower virus transmission rates. Viraemia was low or undetectable in animals treated with astodrimer sodium 1% or infected with treated virus, correlating with the lack of detectable viral replication in the liver. Similarly, low virus replication in the nasal cavity after treatment with astodrimer sodium 1% potentially protected the brain from infection. Astodrimer sodium 1% significantly reduced the pro-inflammatory cytokines IL-6, IL-1α, IL-1β, TNFα and TGFβ and the chemokine MCP-1 in the serum, lung and trachea vs. PBS. Astodrimer sodium 1% nasal spray blocked or reduced SARS-CoV-2 replication and its sequelae in K18-hACE2 mice. These data indicate a potential role for the product in preventing SARS-CoV-2 infection or for reducing the severity of COVID-19.

## 1. Introduction

Severe acute respiratory syndrome coronavirus 2 (SARS-CoV-2) is the airborne viral pathogen that causes the pandemic coronavirus disease of 2019 (COVID-19). The COVID-19 pandemic has resulted in significant worldwide deaths, and the search for strategies to reduce the incidence and clinical severity of the disease continues. The clinical spectrum of COVID-19 ranges from mild, self-limiting respiratory tract illness to severe progressive pneumonia. In addition, many people do not fully recover from the initial respiratory illness and go on to suffer from post-COVID-19 syndrome referred to as long-COVID. The most common symptoms of long-COVID are fatigue, shortness of breath, tightness of the chest, racing heart, difficulty concentrating and brain fog, loss of smell and taste, loss of appetite, hair loss, difficulty sleeping, anxiety and depression [1]. Many of those who suffer from long-COVID had mild initial symptoms and were not hospitalised. The effects of long-COVID are currently associated with a chronic aberrant immune response. There is an urgent need to develop strategies to prevent SARS-CoV-2 infection to eliminate the potential adverse events that infection can cause.

Astodrimer sodium is a large (3–4 nm, ~16.5 kDa), negatively charged, highly-branched dendrimer that has significant antiviral and virucidal activity against different isolates of SARS-CoV-2 in vitro [2]. Its mechanism of action is primarily to act as a barrier to block the binding of the highly positively charged SARS-CoV-2 spike protein to its receptor-complex, which includes the angiotensin converting enzyme 2 (ACE2) receptor. Many viruses, including SARS-CoV-2 [3], utilise negatively charged heparan sulphate proteoglycans (HSPGs) protruding from the cell membrane to potentially bind and guide, or “surf”, the virus to its specific cellular receptor. Astodrimer sodium interferes with these early virus entry steps to block infection with a range of viruses in vitro [4,5] and in vivo [6,7]. The size and negative charge of astodrimer sodium mean that it is not systemically absorbed following topical application to mucosal epithelia [8,9].

SARS-CoV-2 receptors and coreceptors have been shown to be highly expressed in nasal epithelial cells [10], such that intranasally administered therapeutic modalities could be effective in helping to prevent spread of infection of the virus. Astodrimer sodium has been formulated as a nasal spray with the intention of being applied in the nasal cavity to help reduce exposure to infectious viral load, thereby helping to protect from infection with SARS-CoV-2 and other respiratory viruses.

Animal models of SARS-CoV-2 replication and pathogenesis provide an opportunity to study aspects of the disease that are not easily investigated in humans. SARS-CoV-2 infection via the nasal inoculation of K18-hACE2 transgenic mice, a previously established model for the investigation of SARS-CoV infection [11], has been shown to cause SARS-CoV-2 viral load-dependent respiratory illness, with a subset of animals developing brain infection that also contributes to death [12]. The tissue distribution of infectious virus and viral genomic RNA confirms the extensive infection of the lung and brain. In a high viral load (10^5^ plaque forming units [PFU] intranasal dose) infection of mice, small amounts of SARS-CoV-2 RNA were also detected in other tissues including the liver, small intestine, heart, spleen, kidney, large intestine and colon, indicating the low-level distribution of the virus via blood. The histological examination of these tissues identified that the liver was the only other tissue that displayed detectable evidence of the disease [12]. Cytokine, chemokine and innate immune transcripts are elevated in the livers of SARS-CoV-2 infected hACE2 mice, which is indicative of an active immune response to a pathogen [12]. Liver enzyme abnormalities are found in up to 50% of COVID-19 patients, and the virus infection of hepatocytes has been described [13].

The current study extends the established antiviral and virucidal activity of astodrimer sodium against SARS-CoV-2 in vitro to evaluate, in a recognised in vivo challenge model of infection, the ability of the compound in a nasal spray formulation to protect against and reduce the severity of SARS-CoV-2 infection via the upper respiratory tract of K18-hACE2 mice.

The results of the study demonstrated that astodrimer sodium 1% nasal spray formulation demonstrated protective effects in K18-hACE2 mice challenged with SARS-CoV-2 infection via the upper respiratory tract, inhibiting SARS-CoV-2 replication and significantly reducing infectious viral load and the production of pro-inflammatory cytokines.

## 2. Materials and Methods

### 2.1. Animals

Transgenic mice expressing human ACE2 (hACE2) under the control of the cytokeratin 18 promoter (K18) were described in McCray et al., 2007 [11]. The K18 promoter primarily directs gene expression in numerous epithelial-lined tissues [14] and neurons [15]. The SARS-CoV-2 infection of the K18-hACE2 mice has been described in the literature [16,17,18]. These mice are congenic on the C57BL/6 background.

Six-to-eight-week-old K18-hACE2 mice (male or female) were purchased from The Jackson Laboratory (Bar Harbor, ME, USA; Stock No. 034860) and assessed for ill health upon arrival. The animals underwent acclimatisation for 1–2 weeks and were housed individually to minimize the risk of cross infection. The mice were kept by the Department of Animal Resources at The Scripps Research Institute (TSRI) (La Jolla, CA, USA) vivarium and the study was conducted in strict accordance with protocols approved by TSRI Ethics Committee, the Institutional Animal Care and Use Committee (Protocol Number: 20-0007, approved 12 June 2020), and with the recommendations in the Guide for the Care and Use of Laboratory Animals of the National Institutes of Health (NIH).

Animals were maintained under isoflurane anaesthesia for dosing and virus inoculation and were returned to their housing for recovery.

### 2.2. SARS-CoV-2 and Cells

The 2019n-CoV/US-WA1/2020 strain of SARS-CoV-2 was used in these studies (obtained from BEI Resources (Manassas, VA, USA), National Institute of Allergy and Infectious Disease (NIAID), NIH: SARS-Related Coronavirus 2, Isolate USA-WA1/2020, recombinant infectious clone with nanoluciferase gene (icSARS-CoV-2-nLuc), NR-54003). The virus was passaged in Vero E6 cells (CRL-1586™, ATCC, Washington, DC, USA). The Vero E6 cells were maintained in Dulbecco’s Modified Eagle’s Medium (DMEM) supplemented with 10% foetal bovine serum (FBS).

The quantitation of viral inoculum generated in vitro and the amount of virus in tissue homogenates at the end of the study were determined by plaque assay in Vero E6 cells. Viral genomes in serum and tissue homogenates were detected by a quantitative reverse transcriptase polymerase chain reaction (qRT-PCR).

### 2.3. Antiviral and Virucidal Efficacy Studies Experimental Design

#### 2.3.1. Intranasal Administration of Astodrimer Sodium 1% Nasal Spray Formulation

Two groups (Groups 1.1 and 1.2) of three animals each were used to determine the antiviral efficacy of astodrimer sodium 1% nasal spray formulation compared with the placebo (phosphate buffered saline [PBS]), administered via intranasal administration, against SARS-CoV-2 infection in vivo. The animals in Groups 1.1 and 1.2 were inoculated via intranasal administration with 25 µL per nostril of a virus suspension containing 50 PFU/25 µL of SARS-CoV-2 (total challenge: 10^2^ PFU). The animals were treated with PBS (Group 1.1) or 1% astodrimer sodium nasal spray formulation (Group 1.2) via intranasal administration, 25 µL/nostril (total volume of 50 µL), for 7 days. Astodrimer sodium 1% nasal spray or PBS was administered on Day 0, 5 min prior to and 5 min after the virus inoculation, and then once daily on Days 1–6.

#### 2.3.2. Intranasal and Intratracheal Administration of Astodrimer Sodium 1% Nasal Spray Formulation

Two groups (Groups 2.1 and 2.2) of three animals each were used to determine the antiviral efficacy of astodrimer sodium 1% nasal spray formulation compared with placebo (PBS), administered via intranasal and intratracheal administration, against SARS-CoV-2 infection in vivo. The animals in Groups 2.1 and 2.2 were inoculated via intranasal administration with 25 µL per nostril of a virus suspension containing 50 PFU/25 µL of SARS-CoV-2 (total challenge: 10^2^ PFU). The animals were treated with PBS (Group 2.1) or 1% astodrimer sodium nasal spray formulation (Group 2.2) via intranasal (25 µL/nostril, total volume of 50 µL) and intratracheal (2 µL/g body weight) administration for 7 days. Intratracheal administration was performed by inserting a catheter down the mouth and throat. Astodrimer sodium 1% nasal spray or PBS was administered intranasally on Day 0, 5 min prior to and 5 min after virus inoculation, and then once daily on Days 1–6. Astodrimer sodium 1% nasal spray or PBS was administered intratracheally on Day 0, 5 min prior to virus inoculation, and then once daily on Days 1–6.

#### 2.3.3. Virucidal Intervention with Astodrimer Sodium 1% Nasal Spray Formulation

Two groups (Groups 3.1 and 3.2) of three animals each were used to assess the virucidal activity of astodrimer sodium 1% nasal spray formulation and its ability to reduce replication in vivo following the pretreatment of the virus with the product. The virus (3 × 10^2^ PFU) was incubated with 100 µL astodrimer sodium 1% nasal spray formulation or PBS for 60 min at 37 °C. Immediately following the 60 min incubation period, the mixture was pelleted through a 20% sucrose cushion to remove unbound astodrimer sodium and thereby neutralise its effects. The pelleted virus was then resuspended in 50 µL of PBS and administered intranasally (25 µL/nostril). No further product was administered to these animals.

### 2.4. Determination of Endpoints

Body weight and mortality were assessed during the study. The animals were euthanised on Day 7. The status of SARS-CoV-2 infection was determined by measuring the viral load by a qRT-PCR in blood samples (on Day 0, Day 4 and Day 7) and nasal secretions (Day 7), and a qRT-PCR and plaque assay determination of the infectious viral titre of the lung, trachea, brain and liver tissue homogenates (Day 7).

To prepare tissue homogenates, 25 mg of frozen tissue was homogenised using a Bead Genie homogeniser (Scientific Industries, Bohemia, NY, USA). The tissue homogenates were transferred into pre-filled 2.0 mL tubes with stainless steel (acid-washed) homogeniser beads, Ø:2.8 mm (Stellar Scientific, Baltimore, MD, USA) and 500 µL lysis buffer with protease inhibitors. The tubes were shaken at a speed of 6 m/s for 25 s.

The serum and tissue homogenates were analysed for the highly conserved SARS-CoV-2 nucleocapsid RNA by a qRT-PCR using the method previously described by Winkler et al., 2020 [18]. Total viral RNA was extracted from serum, nasal secretions or tissue using the MagMax™ mirVana™ Total RNA Isolation Kit (ThermoFisher Scientific, Waltham, MA, USA) on the KingFisher™ Flex extraction robot (ThermoFisher Scientific, Waltham, MA, USA). The SARS-CoV-2 nucleocapsid (N) gene was reverse transcribed and amplified using the TaqMan^®^ RNA-to CT™ 1-Step Kit (ThermoFisher Scientific, Waltham, MA, USA). The SARS-CoV-2 N gene was detected using Forward primer: ATGCTGCAATCGTGCTACAA; Reverse primer: GACTGCCGCCTCTGCTC; Probe: /56-FAM/TCAAGGAAC/ZEN/AACATTGCCAA/3IABkFQ/. This region was included in an RNA standard to allow for copy number determination down to 10 copies per reaction. The reaction mixture contained final concentrations of primers and probe of 500 and 100 nM, respectively. The reverse transcription was performed at 48 °C for 15 min followed by 2 min at 95 °C. The amplification was achieved over 50 cycles by the following repeated cycle of 95 °C for 15 s and 60 °C for 1 min.

The quantitation of the infectious virus in the tissue homogenates was performed by plaque assay, as described in Paull et al., 2021 [2], with Vero E6 cells as the detecting cell line.

The level of inflammation in the tissues was measured by determining the number of selected cytokines and the amount of interleukin (IL)-6 (RayBio^®^ Mouse IL-6 ELISA[ELM-IL6-1], RayBiotech Life, Peachtree Corners, GA, USA), IL-1 alpha (IL-1α) (RayBio^®^ Mouse IL-1α ELISA [ELM-IL1a-1]), IL-1 beta (IL-1β) (RayBio^®^ Mouse IL-1β ELISA [ELM-IL1b-1], RayBiotech Life, Peachtree Corners, GA, USA), tumour necrosis factor alpha (TNFα) (RayBio^®^ Mouse TNF-alpha ELISA [ELM-TNFa-1], RayBiotech Life, Peachtree Corners, GA, USA), transforming growth factor beta (TGFβ) (TGF-beta-1 Mouse ELISA kit [BMS608-4], ThermoFisher Scientific, Waltham, MA, USA) and monocyte chemoattractant protein (MCP)-1 (also referred to as chemokine [C-C motif] ligand 2 [CCL2]) (RayBio^®^ Mouse MCP-1 ELISA [ELM-MCP1-1], RayBiotech Life, Peachtree Corners, GA, USA) according to their respective manufacturers’ instructions. Cytokines and chemokine were detected in serum samples collected on Days 0, 4 and 7. Day 7 tissue homogenates from trachea and lung were also used to determine the amount of cytokine and chemokine present. Briefly, the ELISA protocols were similar in that they utilised a solid-phase sandwich ELISA design. A cytokine/chemokine target antibody had been precoated to the plate. The samples were added to the wells to bind to the capture antibody. The addition of a second antibody enabled the detection of the target-antibody sandwich complex, which was quantitated using a colorimetric reporting signal that was directly proportional to the concentration in the original specimen.

### 2.5. Statistical Analysis

The means and standard deviations (SD) or standard errors of the mean (SEM) were calculated using GraphPad Prism 9 (GraphPad Software, San Diego, CA, USA). The blood viral load and cytokine/chemokine data were analysed by a two-way analysis of variance (ANOVA) with Bonferroni multiple comparisons (GraphPad Prism 9, GraphPad Software, San Diego, CA, USA). The swab and tissue viral load, infectious viral titre and tissue cytokine/chemokine data were analysed by paired *t*-tests (GraphPad Prism 9, GraphPad Software, San Diego, CA, USA). Significance was established at the two-sided significance level of 0.05.

## 3. Results

### 3.1. Body Weight and Mortality

All mice in both antiviral and virucidal studies survived the infection and were euthanised as planned on Day 7. Animal behaviour was not altered significantly after virus infection or dosing with the tested products over the seven-day observation period. There were no differences between the groups in terms of body weight change.

### 3.2. Time-Course of Viraemia

**Figure 1:**
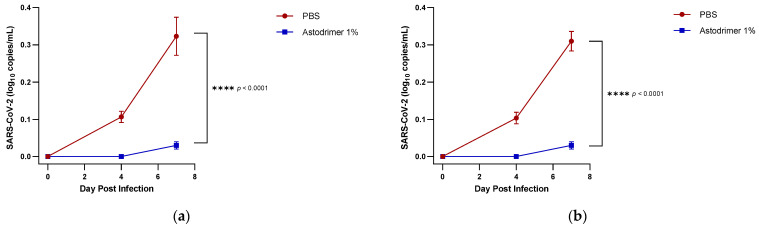
A seven-day time course of viraemia in SARS-CoV-2 (USA-WA1/2020) infected K18-hACE2 mice treated with PBS or astodrimer sodium 1% nasal spray formulation: (**a**) Groups 1.1 and 1.2, intranasal administration only; (**b**) Groups 2.1 and 2.2, intranasal and intratracheal administration. Viraemia was detected by a qRT-PCR for SARS-CoV-2. Points and error bars represent means ± SD.

In antiviral studies, viraemia was detected in the serum of PBS treated Groups 1.1 and 2.1 animals on Day 4 post-infection and increased to ~0.3 log_10_ viral genome copies/mL on Day 7 post-infection. In contrast, animals in Groups 1.2 and 2.2 that were treated with astodrimer sodium 1% nasal spray formulation had undetectable levels of viraemia on Day 4 post-infection, and while viraemia was detectable on Day 7 post-infection, the level was very low (~0.03 log_10_ viral genome copies/mL) (Figure 1). The differences between the viraemia in PBS and the astodrimer sodium 1% nasal formulation treated animals were statistically significant (*p* < 0.0001, two-way ANOVA). There was no significant difference in the amount of viraemia detected when PBS or astodrimer sodium 1% nasal spray formulation were delivered via the intranasal route only or via the intranasal route combined with intratracheal dosing.

**Figure 2:**
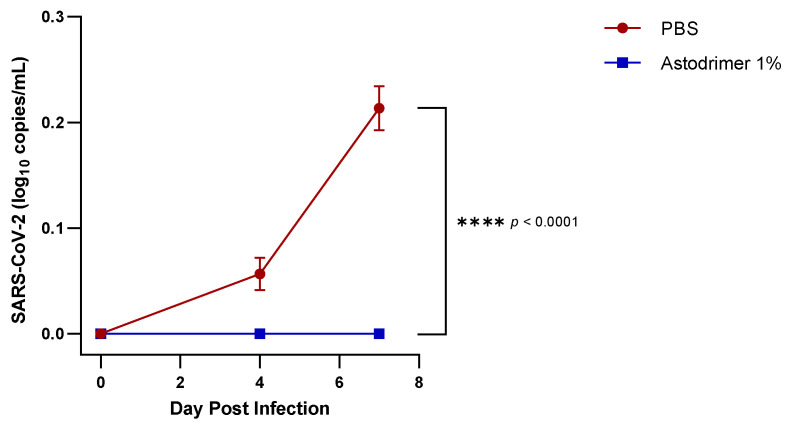
A seven-day time course of viraemia in K18-hACE2 mice infected with SARS-CoV-2 (USA-WA1/2020) inoculum incubated with PBS or astodrimer sodium 1% nasal spray for 60 min prior to the neutralisation procedure—virucidal evaluation. Viraemia was detected by a qRT-PCR for SARS-CoV-2. Points and error bars represent means ± SD.

The PBS control group (Group 3.1) in the virucidal study had detectable viraemia on Days 4 and 7 post-infection, consistent with levels detected in the PBS control Groups 1.1 and 2.1 in the antiviral studies. In contrast, the virucidal study inoculum, consisting of 3 × 10^2^ PFU of virus that had been incubated with 100 µL astodrimer sodium 1% nasal spray formulation for 60 min and followed by the neutralisation of astodrimer sodium activity, resulted in no detectable viraemia on Days 4 or 7 post-infection of any animal in Group 3.2, and the difference with PBS was statistically significant (*p* < 0.0001, two-way ANOVA) (Figure 2).

These data demonstrate that exposure of the virus to astodrimer sodium 1% nasal formulation significantly reduces infectious virus load to a level that was not able to support robust viral replication in an animal model that is ordinarily highly susceptible to virus infection and replication.

### 3.3. Viral Load—Nasal Secretion

Samples of nasal secretion were taken on Day 7 using nasal swabs. The viral titre in the nasal secretions was determined by a qRT-PCR. The expression of the hACE2 receptor in K18-hACE2 mice is high and has been found to support active SARS-CoV-2 replication.

**Figure 3:**
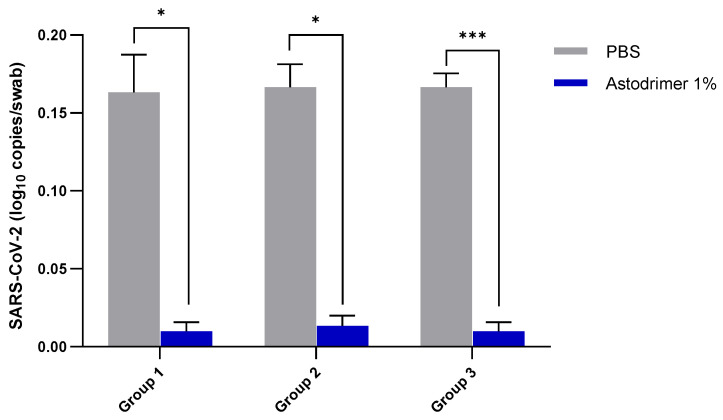
The number of SARS-CoV-2 (USA-WA1/2020) viral genome copies (qRT-PCR) on Day 7 per nasal swab from K18-hACE2 mice treated with PBS or astodrimer sodium 1% nasal spray formulation: Group 1, intranasal administration only; Group 2, intranasal and intratracheal administration; and Group 3, virucidal evaluation. The first (grey) column of each matched data set is the group treated with PBS and the second (blue) column is the group treated with astodrimer sodium 1% nasal spray. Columns and error bars represent mean ± SEM. * *p* < 0.05, *** *p* < 0.001, paired *t*-tests.

In both antiviral groups and the virucidal group, the level of detectable viral genomes in the nasal cavity of animals in the astodrimer sodium 1% nasal spray formulation group was significantly lower than that in the PBS-control group (antiviral groups, *p* < 0.05; virucidal group *p* < 0.001, *t*-test) (Figure 3).

The very low viral genome copy number detected in the nasal secretions of animals treated with 1% astodrimer sodium in Groups 1.2 and 2.2 represents low virus replication and/or potentially fewer cells infected in these nasal swabs. This reduction in viral shedding in the antiviral intervention groups demonstrates that 1% astodrimer sodium treatment in the nasal cavity, with or without intratracheal dosing, can significantly reduce viral shedding and that this reduction may translate to a reduced capacity for transmission.

The single exposure of the virus to astodrimer sodium 1% nasal spray formulation for 60 min in the virucidal study (Group 3.2) did not result in the absence of the virus detected in nasal secretions but did result in a lack of viraemia compared to the PBS control (Group 3.1). In vitro virucidal studies by our group demonstrated a reduction in viral titre of up to 99.99% when 1% astodrimer sodium was used to block SARS-CoV-2 (USA-WA1/2020) infection [2]. Whilst the current virucidal in vivo study demonstrates partial protection from exposure to 3 × 10^2^ PFU SARS-CoV-2 (USA-WA1/2020), the results suggest at least one virus escaped and established a very low-grade infection.

### 3.4. Viral Load—Whole Tissue Homogenate

The K18-hACE2 mouse model has been reported to support virus replication primarily in the respiratory tract and brain and to a lesser extent in other organs such as the liver. Pathogenesis studies with SARS-CoV-2 infection have identified that the mice are highly susceptible to death related to injury to the lung and, in some cases, the brain.

**Figure 4:**
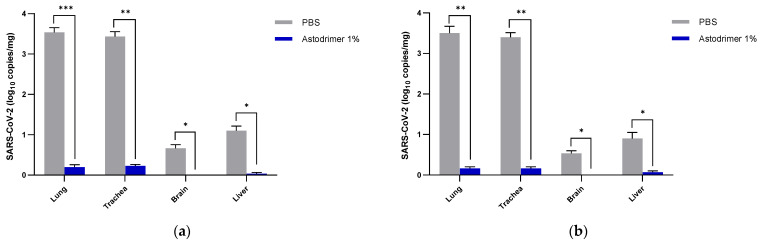
The number of viral genome copies (qRT-PCR) per mg of lung, trachea, brain and liver tissue from SARS-CoV-2 (USA-WA1/2020) infected K18-hACE2 mice treated with PBS or astodrimer sodium 1% nasal spray formulation: (**a**) Groups 1.1 and 1.2, intranasal administration only; (**b**) Groups 2.1 and 2.2, intranasal and intratracheal administration. Tissue collected on Day 7. The first (grey) column of each matched data set is the group treated with PBS and the second (blue) column is the group treated with astodrimer sodium 1% nasal spray. Columns and error bars represent means ± SEM. * *p* < 0.05, ** *p* < 0.01, *** *p* < 0.001, paired *t*-tests.

Viral load was determined in the lung, trachea, brain and liver tissue homogenates by the number of viral genome copies (qRT-PCR) and the amount of infectious virus (plaque assay). In Group 1 and 2 animals, the number of detectable viral genome copies/mg in the lung and trachea were significantly reduced at Day 7 by >3 log (>99.9%) in the animals treated with astodrimer sodium 1% nasal spray (Group 1.2 and 2.2) compared to the PBS treated animals (Group 1.1 and 2.1, respectively) (*p* < 0.01, *t*-test) (Figure 4).

**Figure 5:**
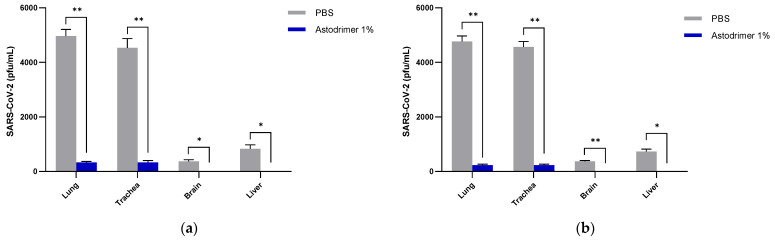
The amount of infectious virus (pfu) per mL of lung, trachea, brain and liver tissue homogenate from SARS-CoV-2 (USA-WA1/2020) infected K18-hACE2 mice treated with PBS or astodrimer sodium 1% nasal spray formulation: (**a**) Groups 1.1 and 1.2, intranasal administration only; (**b**) Groups 2.1 and 2.2, intranasal and intratracheal administration. The first (grey) column of each matched data set is the group treated with PBS and the second (blue) column is the group treated with astodrimer sodium 1% nasal spray. Columns and error bars represent means ± SEM. * *p* < 0.05, ** *p* < 0.01, paired *t*-tests.

Infectious virus in the lung and trachea was also significantly reduced by up to 95% in the astodrimer sodium groups (Groups 1.2 and 2.2) compared to the PBS treated animals (Groups 1.1 and 2.1, respectively) (*p* < 0.01, *t*-test) (Figure 5).

It was most remarkable that there was no detectable infectious virus recovered from the brain or liver in any astodrimer sodium-treated animal of Group 1.2 and 2.2 on Day 7 (Figure 4 and Figure 5).

**Figure 6:**
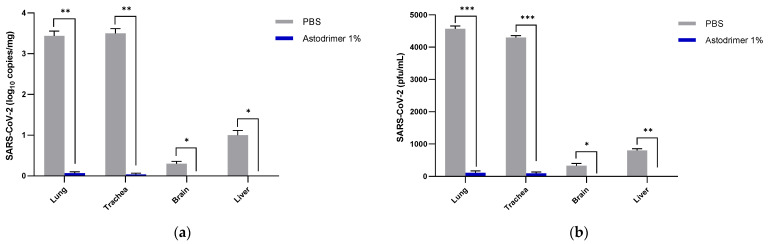
The amount of virus in the K18-hACE2 mice infected with SARS-CoV-2 (USA-WA1/2020) inoculum incubated with PBS (Group 3.1) or astodrimer sodium 1% nasal spray (Group 3.2) for 60 min prior to the neutralisation procedure—virucidal evaluation: (**a**) number of viral genome copies (qRT-PCR) per mg of lung, trachea, brain and liver tissue; (**b**) amount of infectious virus (pfu) per mL of lung, trachea, brain and liver tissue homogenate. The first (grey) column of each matched data set is the group treated with PBS and the second (blue) column is the group treated with astodrimer sodium 1% nasal spray. Columns and error bars represent means ± SEM. * *p* < 0.05, ** *p* < 0.01, *** *p* < 0.001, paired *t*-tests.

In the virucidal Group 3 animals, the number of detectable viral genome copies/mg in the lung and trachea was significantly reduced by >3 log (>99.9%) in the animals infected with 1% astodrimer sodium-treated SARS-CoV-2 (Group 3.2) compared to the animals infected with PBS-treated SARS-CoV-2 (Group 3.1) (*p* < 0.01, *t*-test). Infectious virus in the lung and trachea was also significantly reduced by >99% in the astodrimer sodium group (Group 3.2) compared to the PBS animals (Group 3.1) (*p* < 0.001, *t*-test) (Figure 6). Again, it was remarkable that there was no detectable viral genome copies or infectious virus detected in the brain or liver in any animal of Group 3.2 on Day 7 (Figure 6a,b, respectively).

### 3.5. Pro-Inflammatory Cytokines and Chemokine Detection in Serum, Trachea and Lung Homogenates

**Figure 7:**
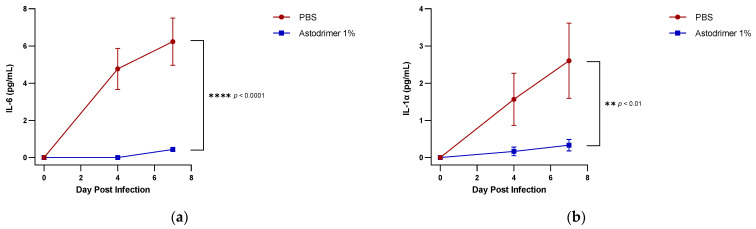

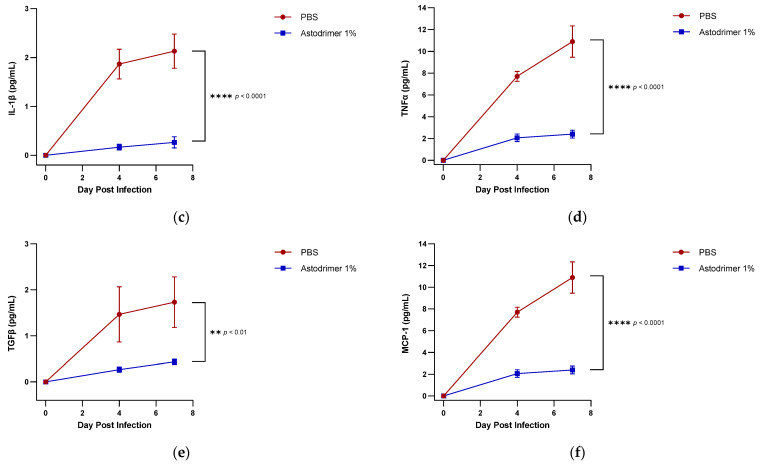
A seven-day time course of the amount of cytokine/chemokine (pg/mL) in the serum of SARS-CoV-2 (USA-WA1/2020) infected K18-hACE2 mice treated with PBS or astodrimer sodium 1% nasal spray formulation via intranasal administration only (Groups 1.1 [PBS] and 1.2): (**a**) IL-6 (**b**) IL-1α (**c**) IL-1β (**d**) TNFα (**e**) TGFβ (**f**) MCP-1. Points and error bars represent means ± SD.

There was no detection of cytokines (IL-6, IL-1α, IL-1β, TNFα and TGFβ) or chemokine (MCP-1) in any Day 0 sample from any animal. Following the intranasal infection with 10^2^ (Groups 1.1 and 2.1) or 3 × 10^2^ (Group 3.1) PFU of SARS-CoV-2, there was a time-dependent increase in pro-inflammatory IL-6, IL-1α, IL-1β, TNFα, TGFβ and MCP-1 pg/mL in serum over the 7 days of the experiment (Group 1 data: Figure 7; Groups 2 and 3 data: Figures S1 and S2, respectively). This increase in these pro-inflammatory cytokines and chemokine is indicative of the host’s immune response to the viral infection. In contrast, all animals in Groups 1.2, 2.2 and 3.2 had statistically significantly lower levels (~10 times) of systemic cytokine and chemokine levels on Day 7 (Group 1 data: Figure 7; Groups 2 and 3 data: Figures S1 and S2, respectively). These data, combined with the lower viral load in the serum, suggest that the presence of 1% astodrimer sodium significantly retarded viral infection and dissemination throughout the bodies of these animals. The presence of astodrimer sodium 1% reduced systemic pro-inflammatory cytokines and chemokine in both the antiviral and virucidal studies in vivo.

**Figure 8:**
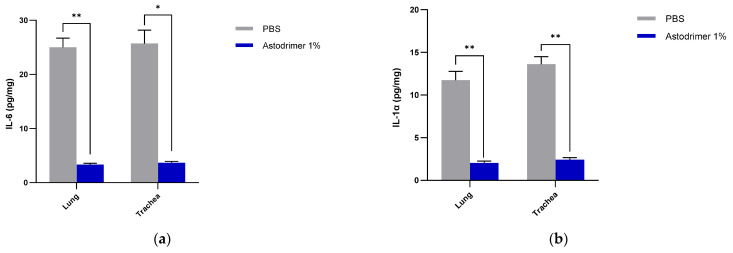

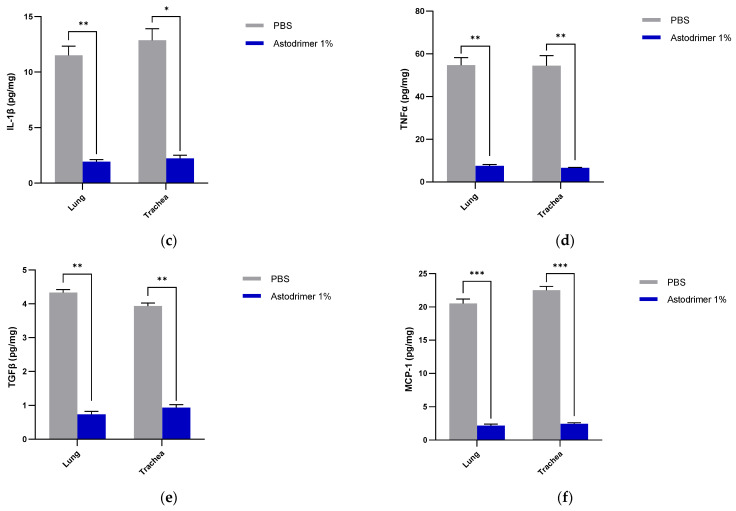
The amount of cytokine/chemokine in the lung and trachea tissue homogenates from K18-hACE2 mice infected with SARS-CoV-2 (USA-WA1/2020) inoculum incubated with PBS (Group 3.1) or astodrimer sodium 1% nasal spray (Group 3.2) for 60 min prior to the neutralisation procedure—virucidal evaluation: (**a**) IL-6 (**b**) IL-1α (**c**) IL-1β (**d**) TNFα (**e**) TGFβ (**f**) MCP-1. The first (grey) column of each matched data set is the group treated with PBS and the second (blue) column is the group treated with astodrimer sodium 1% nasal spray. Columns and error bars represent means ± SEM. * *p* < 0.05, ** *p* < 0.01, *** *p* < 0.001, paired *t*-tests.

The lung and trachea homogenates from all animals had detectable cytokines (IL-6, IL-1α, IL-1β, TNFα and TGFβ) and chemokine (MCP-1) at Day 7 post-infection. In this study, we did not include age-matched uninfected animals to determine the natural range of cytokine production in these tissues. The data from all three groups show the same trend as serum, in that the astodrimer sodium 1% nasal spray formulation used as an antiviral (Figures S3 and S4) or virucidal agent (Figure 8) results in a statistically significant reduction of all of the selected pro-inflammatory cytokines and chemokine in the lungs and trachea.

In the virucidal Group 3.2, there were detectable levels of all five cytokines and one chemokine in the serum by Day 7 post-infection of the study (Figure S2). On Day 4 post-infection there were no or very low detectable levels of systemic IL-6, IL-1α or IL-1β in the Group 3.2 animals (Figure S2). Interestingly, there was no detectable viral load in the serum of any animal at either Day 4 or Day 7 post-infection in Group 3.2 (Figure 1). Very low levels of infectious virus were detectable in the lung and trachea of animals in Group 3.2, indicating that these animals were infected with the virus (Figure 6b). These data suggest that the detection of selected systemic cytokines/chemokine (TNFα, TGFβ and MCP-1) may be more sensitive to the detection of viral infection than of viral load at Day 4 post-infection and, in addition to IL-6, IL-1α and IL-1β, at Day 7 post-infection.

## 4. Discussion

The transmission of SARS-CoV-2 occurs via exposure to airborne infectious respiratory fluids released during exhalation [19]. The amount of SARS-CoV-2 necessary to establish human infection is unknown, and exposure amounts vary based on the infectivity of the carrier and on the environmental conditions. The average viral titre in the saliva of an infectious individual was calculated as 7 × 10^6^ viral RNA copies/mL [20] and has been reported to be as high as 10^11^ viral RNA copies/mL [21]. However, virus particles in the air were calculated to be approximately 40 viral RNA copies per m^3^ in medical staff areas [22]. The actual viral load a susceptible human may receive is difficult to estimate. Mathematical modelling suggests that the number of SARS-CoV-2 particles needed to infect an individual is the equivalent of ~10 to 100 PFU. Due to the absence of specific data for SARS-CoV-2, this number was based on comparisons of infectious doses with other coronaviruses, including SARS-CoV-1, and influenza [23]. The infectious doses of SARS-CoV-2 used in the current K18-hACE2 mice studies are comparable to N*_inf_* used in mathematical modelling for the possible transmission of SARS-CoV-2.

However, the amount of virus given to animal models of SARS-CoV-2 is probably significantly higher than a human infectious dose and is delivered to establish a reproducible infection process in order to study virus replication and pathogenesis. The K18-hACE2 mouse model was chosen for these antiviral studies because it is a model of high SARS-CoV-2 replication and, in certain conditions, is a lethal model of SARS-CoV-2 infection. In the current study, we have achieved viral infection of the lung via intranasal dosing. Astodrimer sodium 1% is currently formulated in a nasal spray and its antiviral and virucidal against SARS-CoV-2 virus replication and pathogenesis were examined.

In these studies, the intranasal administration of astodrimer sodium 1% nasal spray as an antiviral or virucidal agent reduced viral replication in the serum, lung, trachea, brain and liver and the production of pro-inflammatory cytokines (IL-6, IL-1α, IL-1β, TNFα and TGFβ) and chemokine MCP-1 in the serum, lung and trachea at 7 days post-infection. While the complete prevention of infection was not achieved with antiviral or virucidal treatment with astodrimer sodium 1% nasal spray, the evidence supports a significant reduction in SARS-CoV-2 replication and pro-inflammatory cytokines. The severity of COVID-19 has been related to the development of a cytokine storm that is initiated and sustained by pro-inflammatory signaling that leads directly to disease progression. The experiments being reported here support the hypothesis that the intranasal administration of astodrimer sodium 1% nasal spray reduces systemic, lung and tracheal pro-inflammatory cytokine production caused by SARS-CoV-2 infection via the nasal passages. Numerous articles have described the cytokine storm related to COVID-19, but they have not always reported on the same inflammation markers. IL-6, MCP-1, TNFα and other immune markers, particularly those involved in myeloid cell recruitment and function, have been identified as markers that are important in the development of COVID-19 [24,25,26].

A critique of the K18-hACE2 mouse model has been that untreated mice have higher SARS-CoV-2 infection levels of the brain and may die from brain encephalitis rather than pneumonia. In the antiviral and virucidal studies reported here, there was no detectable infectious virus in the brain when they were treated with astodrimer sodium 1% intranasally +/− intratracheal dosing. Studies performed on the autopsies of people who died due to COVID-19 have identified virus genomes, proteins and particles in the brain [27]. One theory is that the brain is infected by virus transmitted via neurons in the olfactory bulb, located in upper nasal cavity, to the brain. Another is that it can infect the brain hematogeneously [28,29]. The intranasal +/− intratracheal administration of astodrimer sodium 1% nasal spray in the antiviral studies and the single virucidal dose of astodrimer sodium 1% efficiently blocks this route of viral spread, and these data may translate to protection from brain infection and its sequelae in humans, such as many of the symptoms of long-COVID.

The significant reduction in viral replication in the liver is an important finding. In up to 50% of COVID-19 cases, liver enzymes, which are used to assess liver damage, are elevated [30]. The liver is presumably infected via the blood, and liver infection has been associated with clotting abnormalities in humans infected with SARS-CoV-2. A reduction in the virus in this organ in Groups 1.2, 2.2 and 3.2 is likely related to lower levels of disseminating virus (Figure 1) that have reached the liver.

Although the number of animals per group is limited in this study, highly statistically significant reductions in viral load were observed. The combined data over the three groups are supportive of astodrimer sodium 1% nasal spray inactivating virus and blocking SARS-CoV-2 infection, resulting in significantly lower viral loads and infectious virus in the lung, trachea, brain and liver of K18-hACE2 mice. Astodrimer sodium 1% intranasal treatment as an antiviral or as a virucidal agent significantly reduced the titre of virus in all tissues assessed. While the antiviral and virucidal interventions did not completely prevent infection in this highly challenging model, the amount of virus that escaped the intervention was significantly lower, as determined by the viral load in serum and tissues. Furthermore, there was evidence of reduced virus shedding from the nasal cavity and transmission of the virus to the brain, presumably via olfactory bulb neurons and/or blood, as well as the infection of the liver, presumably via blood.

While this study did not investigate the toxicity of astodrimer sodium 1% nasal spray, there were no signs of toxicity observed in the K18-hACE2 mice. The safety of astodrimer sodium 1% nasal spray has been investigated separately in regulatory compliant nonclinical studies and determined to be non-irritant following repeated dosing, non-cytotoxic and non-sensitising. Astodrimer sodium was not systemically absorbed after nasal administration, so the risk of systemic toxicity is negligible. A clinical investigation in healthy volunteers has also recently been completed and showed the product to be well tolerated after dosing four times daily for 14 days, with full data to be published.

Current therapies for the prevention of COVID-19 are dominated by vaccine strategies that provide a reduction in the severity of the disease rather than absolute protection from infection and the ability to shed and transmit the virus. Complementary interventions will continue to be necessary to reduce the transmission of the virus, particularly in the current environment where the dominant variants of SARS-CoV-2 have higher transmission rates. Given the protective effects against a clinical isolate of SARS-CoV-2 observed in the current study in K18-hACE2 mice, as well as the favourable safety profile in nonclinical and clinical studies, astodrimer sodium 1% nasal spray has the potential for personal use in humans to help protect from the aggressive spread of SARS-CoV-2 infection, and it may reduce the severity or frequency of respiratory, central nervous system and gastrointestinal clinical outcomes of infection.

## 5. Conclusions

The combined data indicate that astodrimer sodium 1% nasal spray inactivates the virus, and the resulting reduction in the exposure to the virus in vivo limits SARS-CoV-2 infection, resulting in significantly lower viral loads and infectious virus in the lung, trachea, brain and liver of K18-hACE2 mic, and significantly lower pro-inflammatory cytokine production. The study provides evidence that astodrimer sodium 1% nasal spray significantly reduced the severity of SARS-CoV-2 replication and pathogenesis in the highly susceptible K18-hACE2 mouse model. The potential protective effects of astodrimer sodium 1% nasal spray warrant further investigation to help combat SARS-CoV-2 infection in humans.

## Data Availability

All data are included in the manuscript and Supplementary Materials.

## Supplementary Materials

The following are available online at https://www.mdpi.com/article/10.3390/v13081656/s1, Figure S1: A seven-day time course of the amount of cytokine/chemokine (pg/mL) in the serum of SARS-CoV-2 (USA-WA1/2020) infected K18-hACE2 mice treated with PBS (Group 2.1) or astodrimer sodium 1% nasal spray formulation (Group 2.2) via intranasal and intratracheal administration, Figure S2: A seven-day time course of the amount of cytokine/chemokine (pg/mL) in the serum of in K18-hACE2 mice infected with SARS-CoV-2 (USA-WA1/2020) inoculum incubated with PBS (Group 3.1) or astodrimer sodium 1% nasal spray (Group 3.2) for 60 min prior to the neutralisation procedure—virucidal evaluation, Figure S3: The amount of cytokine/chemokine in the lung and trachea tissue homogenates from SARS-CoV-2 (USA-WA1/2020) infected K18-hACE2 mice treated with PBS (Group 1.1) or astodrimer sodium 1% nasal spray (Group 1.2) formulation via intranasal administration, Figure S4: The amount of cytokine/chemokine in the lung and trachea tissue homogenates from SARS-CoV-2 (USA-WA1/2020) infected K18-hACE2 mice treated with PBS (Group 2.1) or astodrimer sodium 1% nasal spray (Group 2.2) formulation via intranasal and intratracheal administration.
