# Supplementary material for: Protective Effects of Astodrimer Sodium 1% Nasal Spray Formulation against SARS-CoV-2 Nasal Challenge in K18-hACE2 Mice
Source: Viruses. 2021 Aug 20;13(8):1656. doi: 10.3390/v13081656 (PMC8402925; doi:10.3390/v13081656)
Supplement: Supplementary file 1 [file viruses-13-01656-s001.zip › viruses-1290079-supplementary.pdf]

viruses

Supplementary Materials

Protective Effects of Astodrimer Sodium 1% Nasal Spray Formulation Against SARS-CoV-2 Nasal Challenge in K18-hACE2 Mice

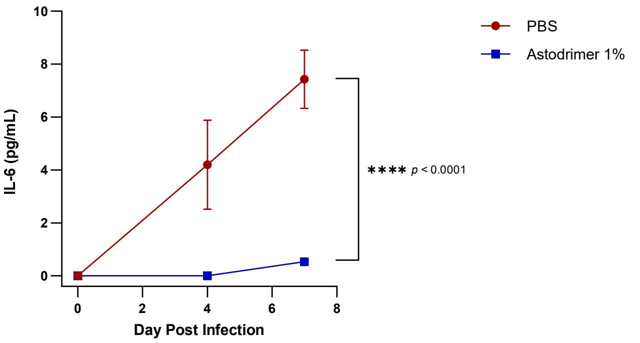

(**a**)

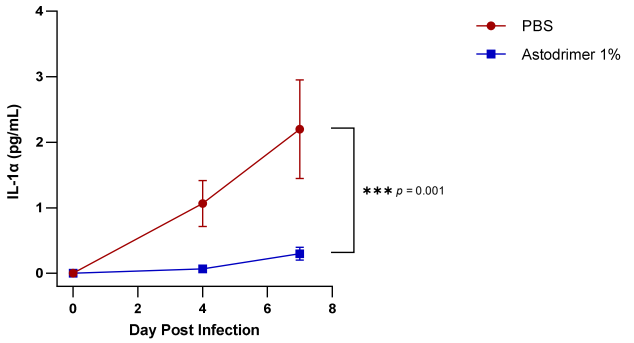

(**b**)

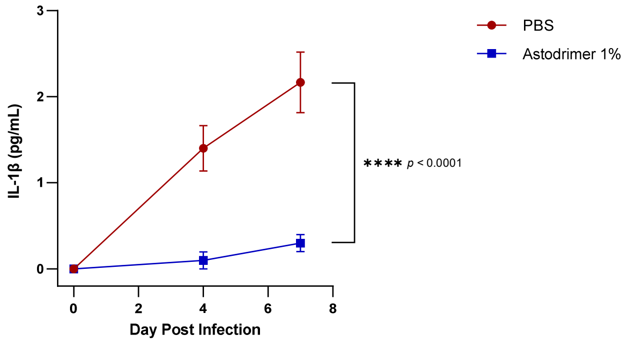

(**c**)

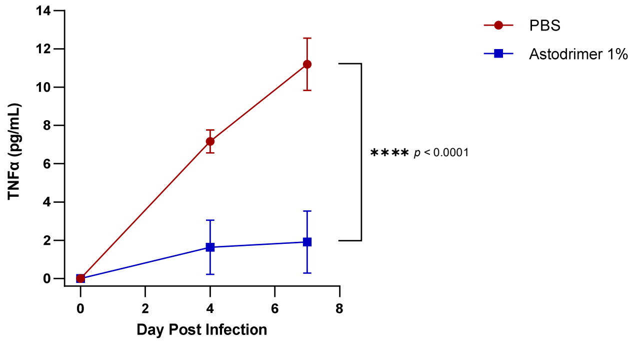

(**d**)

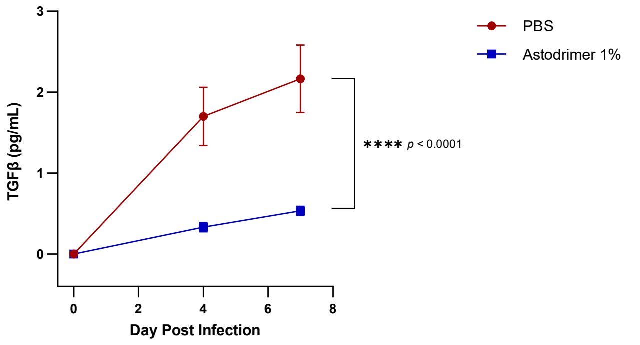

(**e**)

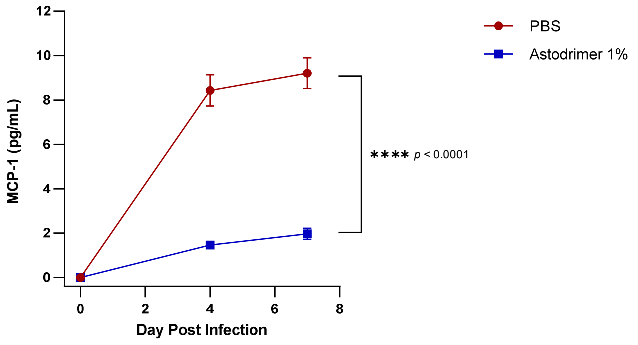

(**f**)

Figure S1. A seven-day time course of amount of cytokine/chemokine (pg/mL) in serum of SARS-CoV-2 (USA-WA1/2020) infected K18-hACE2 mice treated with PBS (Group 2.1) or astodrimer sodium 1% nasal spray formulation (Group 2.2) via intranasal and intratracheal administration: (**a**) IL-6 (**b**) IL-1*α* (**c**) IL-1β (**d**) TNFα (**e**) TGFβ (**f**) MCP-1. Points and error bars represent means ± SD.

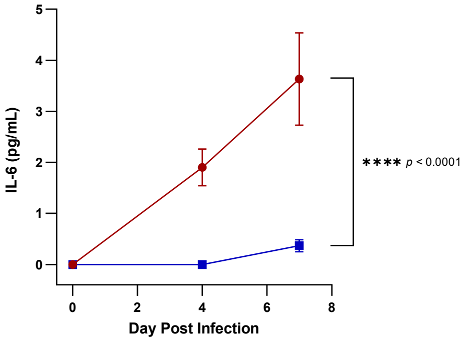

(**a**)

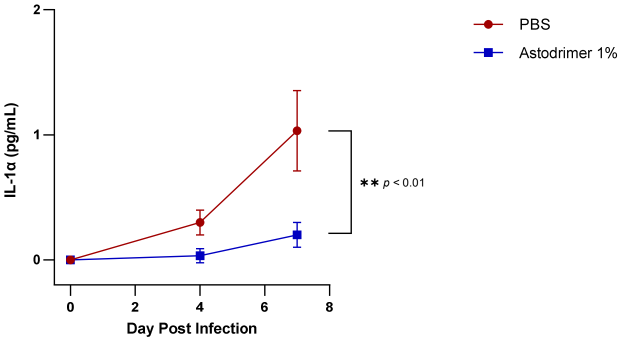

(**b**)

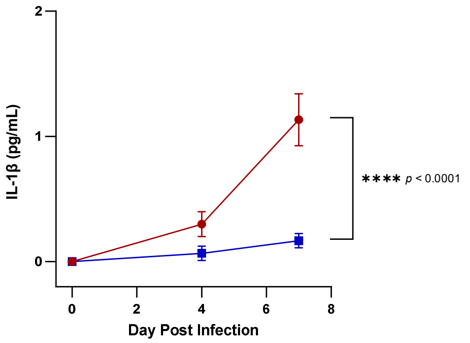

(**c**)

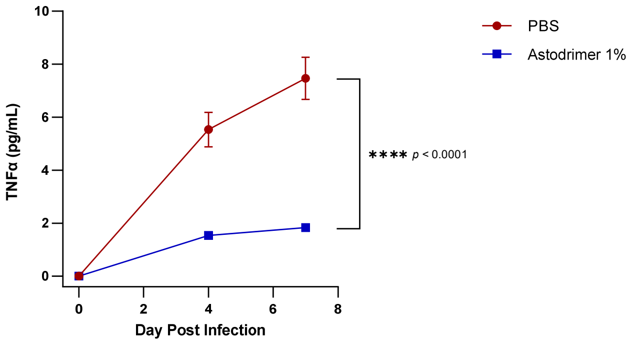

(**d**)

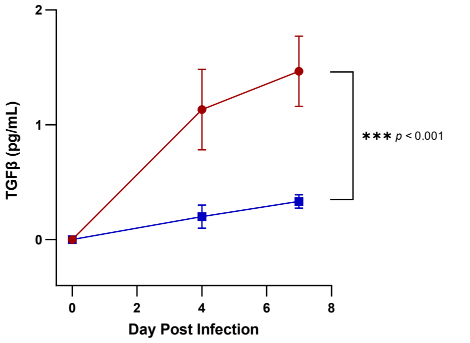

(**e**)

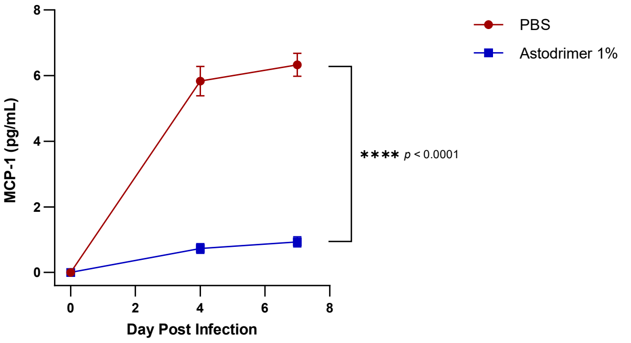

(**f**)

Figure S2. A seven-day time course of amount of cytokine/chemokine (pg/mL) in serum of in K18-hACE2 mice infected with SARS-CoV-2 (USA-WA1/2020) inoculum incubated with PBS (Group 3.1) or astodrimer sodium 1% nasal spray (Group 3.2) for 60 minutes prior to neutralisation procedure – virucidal evaluation: (**a**) IL-6 (**b**) IL-1α (**c**) IL-1β (**d**) TNFα (**e**) TGFβ (**f**) MCP-1. Points and error bars represent means ± SD.

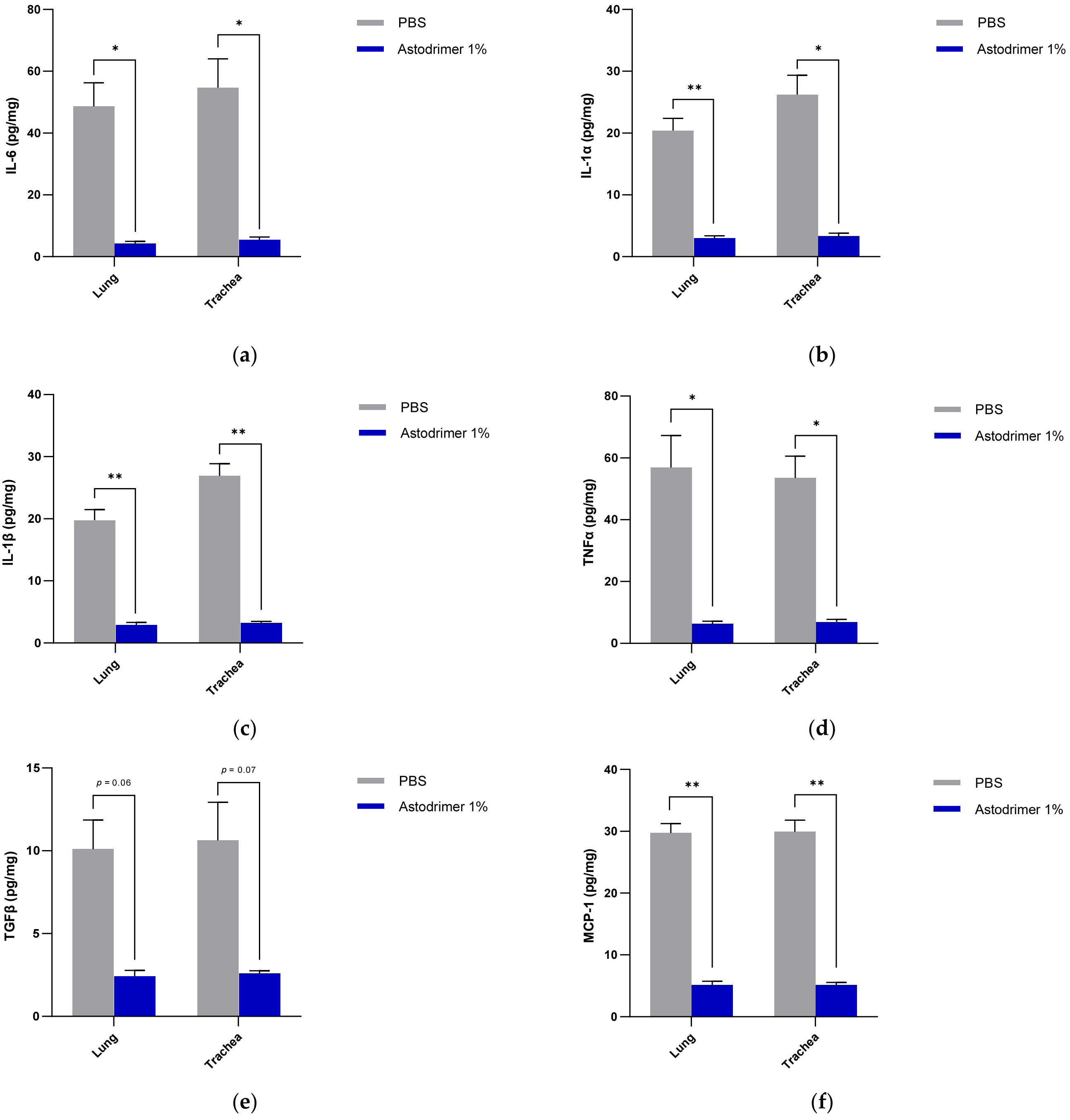

Figure S3. Amount of cytokine/chemokine in lung and trachea tissue homogenates from SARS-CoV-2 (USA-WA1/2020) infected K18-hACE2 mice treated with PBS (Group 1.1) or astodrimer sodium 1% nasal spray (Group 1.2) formulation via intranasal administration: (**a**) IL-6 (**b**) IL-1α (**c**) IL-1β (**d**) TNFα (**e**) TGFβ (**f**) MCP-1. Columns and error bars represent means ± SEM. * $p < 0.05$, ** $p < 0.01$, paired t-tests.

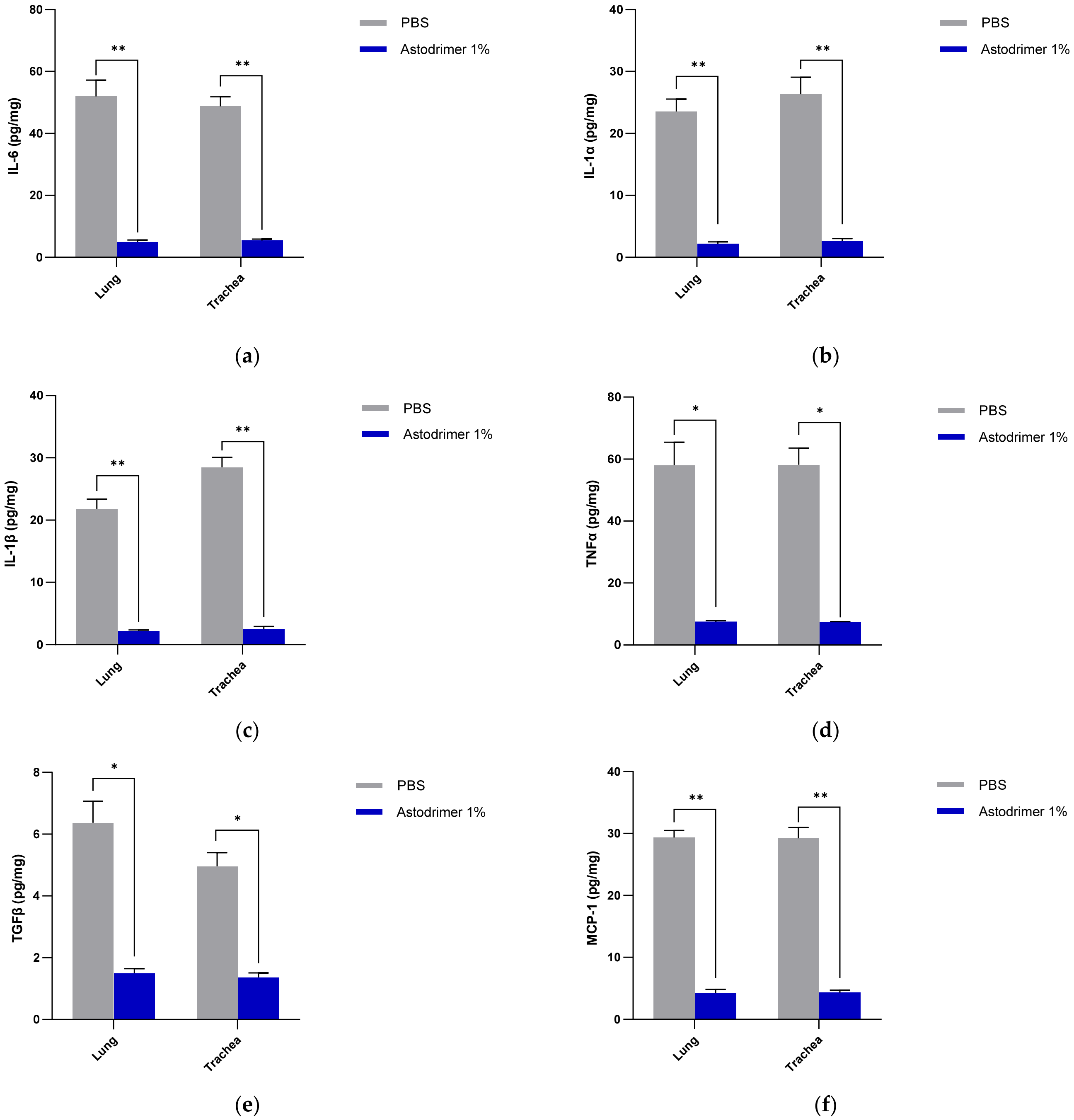

Figure S4. Amount of cytokine/chemokine in lung and trachea tissue homogenates from SARS-CoV-2 (USA-WA1/2020) infected K18-hACE2 mice treated with PBS (Group 2.1) or astodrimer sodium 1% nasal spray (Group 2.2) formulation via intranasal and intratracheal administration: (**a**) IL-6 (**b**) IL-1α (**c**) IL-1β (**d**) TNFα (**e**) TGFβ (**f**) MCP-1. Columns and error bars represent means ± SEM. * $p < 0.05$, ** $p < 0.01$, paired t-tests.
